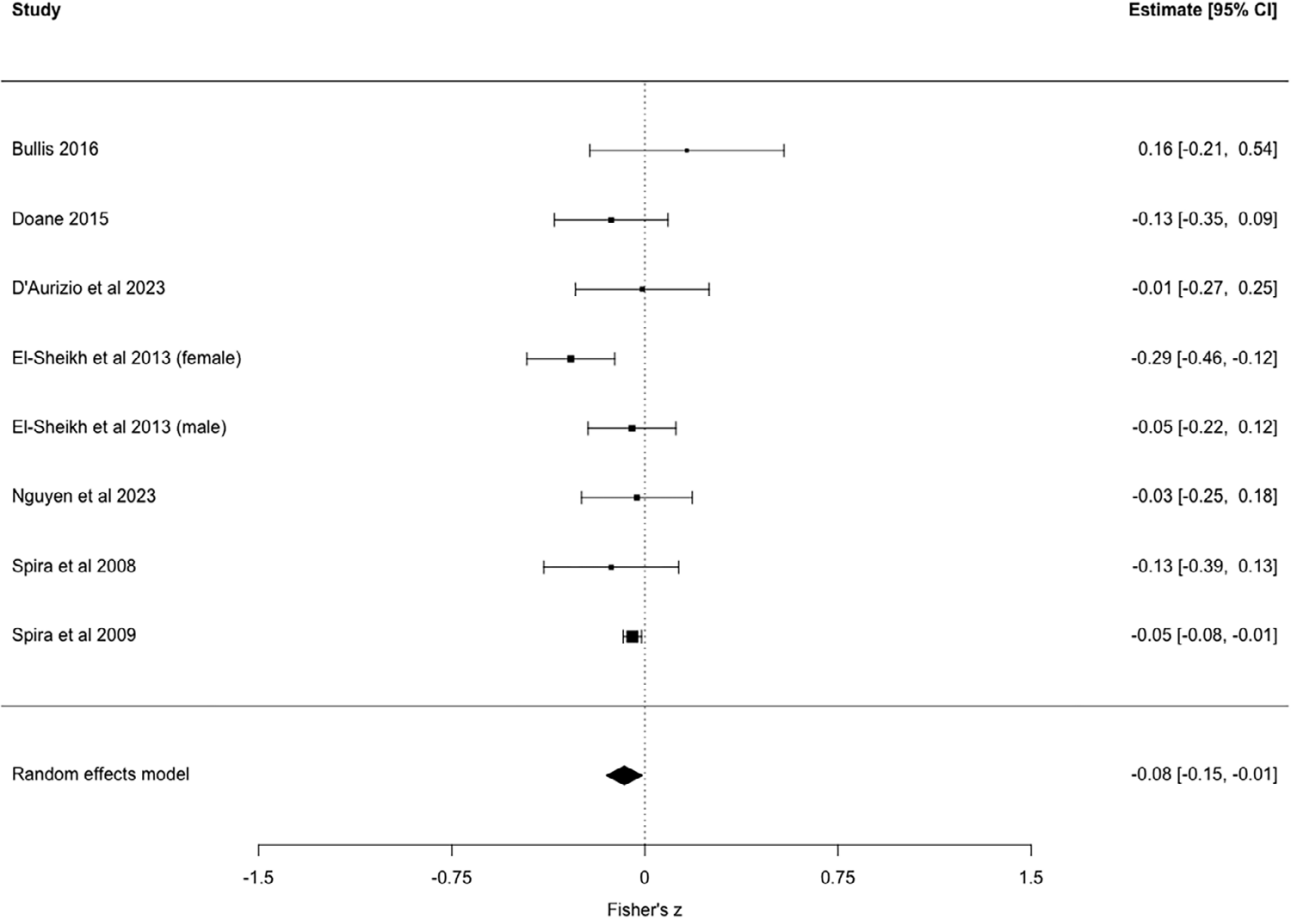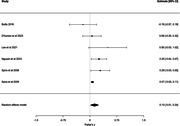# Association between digital biomarkers and anxiety: a systematic review and meta‐analysis

**DOI:** 10.1002/alz.089051

**Published:** 2025-01-09

**Authors:** Yolanda Lau, Natalia Chemas, Heema Ajeet Gokani, Rachel Morrell, Zuzana Walker, Harriet Demnitz‐King, Natalie L Marchant

**Affiliations:** ^1^ Division of Psychiatry, University College London, London UK; ^2^ Unit for Social and Community Psychiatry, East London NHS Foundation Trust, London UK; ^3^ Essex Partnership University NHS Foundation Trust, Wickford UK

## Abstract

**Background:**

Anxiety, both generalised anxiety disorder and anxiety symptoms, has been recognised as a risk factor and prodromal symptom of dementia. Digital biomarkers are gaining interest as proxy markers for mental health because they enable passive and continuous data collection, allowing for early detection. However, the association between digital biomarkers and anxiety remains unknown. This systematic review and meta‐analysis aimed to examine the association between digital biomarkers obtained from wrist‐worn wearables and anxiety symptoms in adults.

**Method:**

Systematic literature searches were conducted across six databases, including unpublished grey literature. Studies investigating the association between digital biomarkers from wrist‐worn wearables and anxiety were eligible for this review. Effect sizes were combined across studies, for each digital biomarker separately, using random‐effects meta‐analyses whenever possible. Sensitivity analyses were performed to assess whether results differed according to anxiety type (state, trait), age group (young, middle, older), and sex.

**Result:**

Twenty‐two articles were eligible. Meta‐analyses were conducted for four sleep metrics: sleep efficiency, wake after sleep onset, total sleep time, and sleep onset latency. Analyses revealed that sleep efficiency (8 studies, Fisher’s z = ‐0.08, 95% confidence interval [CI] = ‐0.15 to ‐0.01, p = 0.0263) and wake after sleep onset (6 studies, Fisher’s z = 0.13, 95% CI = 0.01 to 0.24, p = 0.0291) were associated with anxiety symptoms. In sensitivity analyses, associations persisted older adults (for sleep efficiency only) and trait anxiety. Meta‐analyses could not be conducted for physical activity metrics, however, a qualitative synthesis of the limited number of studies (five studies) revealed inconsistent results.

**Conclusion:**

Worse sleep efficiency and longer wake after sleep onset were associated with greater anxiety symptoms. However, due to the limited number of studies, the association with physical activity remains unclear, warranting further research. Further, it became apparent that machine learning studies in this area are limited and of variable quality. Given anxiety is a prodromal symptom of dementia, future research focusing on older adults is essential to explore the use of digital biomarkers in the context of dementia risk.